# New-Onset Depression Is Associated With Low Income and Adverse Arthroplasty-Related Complications

**DOI:** 10.7759/cureus.79311

**Published:** 2025-02-19

**Authors:** Suin Jeong, Ji Won Lee, Elias K Shaya, Henry Boucher

**Affiliations:** 1 Orthopedics, MedStar Georgetown University Hospital, Washington, USA; 2 Orthopedics, MedStar Union Memorial Hospital, Baltimore, USA; 3 Psychiatry, MedStar Good Samaritan Hospital, Baltimore, USA

**Keywords:** hip and knee replacement, post-operative complication, postoperative depression, socioeconomic factors, total joint arthroplasty

## Abstract

Background and objective

Depression may manifest in total joint arthroplasty (TJA) patients even in the absence of any prior history. While research has demonstrated depression to be associated with low socioeconomic status and TJA complications, no studies have explored these associations related to new-onset depression in post-TJA settings. Hence, this study aimed to address that by conducting a retrospective, database-centric study.

Methods

A national database was queried for data on primary total hip arthroplasty (THA) and total knee arthroplasty (TKA) patients ≥18 years with hip/knee osteoarthritis. Patients with prior depression or antidepressant use, or those with a diagnosis of depression >6 months post-surgery were excluded. The study groups comprised patients with new-onset depression within six months of undergoing TJA; the control groups consisted of those without. Study and controls were matched in a 1:4 ratio in terms of age, sex, and comorbidities. Logistic regressions were employed to assess the associations between new-onset depression and (1) income and (2) one-year complications.

Results

THA and TKA study groups had 1.48 times (95% CI: 1.40, 1.57) and 1.66 times (95% CI: 1.60, 1.73) higher odds of being in the lower-income class than respective controls. THA study group had 1.93 times (95% CI: 1.50, 2.46), 2.53 times (95% CI: 2.11, 3.01), and 1.56 times (95% CI: 1.31, 1.86) higher odds of periprosthetic fracture, prosthetic joint infection, and revision than controls; the corresponding values for the TKA study group were 1.90 times (95% CI: 1.34, 2.67), 2.33 times (95% CI: 2.06, 2.63), and 1.59 times (95% CI: 1.37, 1.85) compared to controls.

Conclusions

Low income is associated with the risk of developing new-onset depression post-TJA. New-onset depression is also associated with increased postoperative complications. Future studies may explore whether antidepressant use is beneficial in this population.

## Introduction

Depression is a common psychiatric comorbidity among patients undergoing total joint arthroplasty (TJA) with an estimated prevalence of over 10% [1]. The presence of depression may negatively impact patients’ recovery and quality of life by impairing their treatment adherence [2] and willingness to participate in rehabilitation [3]. Studies have shown that patients with pre-existing depression experience greater patient dissatisfaction, lower patient-reported outcomes and functionality scores, and higher costs related to increased healthcare utilization (i.e., readmission, non-home discharge, and increased complication rates) compared to non-depressed patients [1,4-7].

New-onset depression, i.e., the development of depressive symptoms in patients who were not diagnosed with depression before surgery, may unexpectedly occur in the postoperative period. As this is unexpected, it can pose a double dilemma: the patients are unaware of depressive symptoms as they arise and underreport them, and clinicians may be unprepared to diagnose and treat depression among patients who experience depressive symptoms. While it is known that a subset of TJA patients with undiagnosed or an absence of depression before surgery can develop depression in the postoperative period [8,9] from increased stress, inflammation, and/or pain [9], we need a better understanding of risk factors and ramifications of new-onset depression. This will enable patients who develop this condition to be (1) informed of depressive symptoms that may arise immediately after surgery and (2) equipped to connect to appropriate clinicians who can best diagnose and manage depression.

Prior studies investigating new-onset depression among TJA patients have identified demographic and medical risk factors for this condition, such as female sex, young age, high comorbidity burden, anxiety, smoking, alcohol or drug use, opioid use, non-home discharge, and hospital readmission within six months [10-12]. However, there is limited evidence on the role of social determinants of health (SDOH), specifically the role of socioeconomic factors (SEFs) such as employment status and income in the development of new-onset depression. SDOH refers to conditions that affect health outcomes, such as education, economic instability, unemployment, and housing and food insecurity [13]. Of note, SEFs affect healthcare access by determining one's ability to afford care, influencing insurance coverage, and shaping access to essential resources like transportation [14]. Understanding SEFs is important due to the higher prevalence of depression among low-income individuals [15] and the potential for underdiagnosis and subsequent undertreatment of depression in this group, which may be due to limited healthcare resources and access [16].

Additionally, not only is understanding the risk factors for new-onset depression important, but it is also important to consider how the condition might impact postoperative results. Such insights would help surgeons determine whether the issue is significant from a surgical standpoint and make timely, appropriate patient referrals. Unlike pre-existing depression, which has been linked to poor short-term postoperative outcomes following TJA [1,4-7], the effect of new-onset depression on postoperative complications, is less clear. Prior studies found short-term postoperative complications to be risk factors for the development of new-onset depression by confirming its presence following complications within one year of TJA [10,11]. However, whether new-onset depression itself is an actual risk factor for postoperative complications as is the case with pre-existing depression [1,4-7] remains unknown.

To address these gaps, we relied on a national administrative claims database to investigate (1) the association between SEFs (i.e., employment status and income) and the development of new-onset depression; and (2) the association between the presence of new-onset depression and the odds of one-year arthroplasty-related complications and revisions.

## Materials and methods

Study design and setting

We conducted a retrospective database analysis involving the PearlDiver Mariner Patient Claims Database (PearlDiver Technologies, Colorado Springs, CO), a Health Insurance Portability and Accountability Act-compliant national database. PearlDiver’s M161Ortho dataset contains de-identified records for over 161 million distinct patients from January 2010 to April 2022. The strength of the PearlDiver database is its ability to allow longitudinal research with unique patient identifier codes, and the latest update of the PearlDiver database added socioeconomic data based upon U.S. Census Bureau findings by year and adjusted to the three-digit ZIP code level. This database also provides the largest sample of patients based on all-payer claims (commercial, Medicare, Medicaid, government, and cash), which are adjudicated and audited by independent third parties.

Sample

We queried the M161Ortho dataset using Current Procedural Terminology (CPT), International Classification of Diseases-Ninth Revision (ICD-9) and Tenth Revision (ICD-10), and Uniform System of Classification (USC) codes to obtain our study population (Appendix A). Patients included those who underwent primary total hip arthroplasty (THA) or TKA, with a diagnosis of hip or knee osteoarthritis, and aged 18 years or older at the time of surgery. Individuals with preoperative depression or antidepressant use, those first diagnosed with depression beyond six months post-surgery (to prevent potential confounding when comparing one-year postoperative complications), and those failing to meet the minimum study follow-up of one year were excluded.

A total of 333,411 primary THA and 649,797 TKA patients were included in the analyses. Of these, patients with a diagnosis of depression within six months of surgery were identified as the study groups (6,848 THA and 15,829 TKA patients). Those without a new postoperative diagnosis of depression served as the control groups (326,563 THA and 633,968 TKA patients). Two sets of study and control groups for THA and TKA patients were respectively matched in a study-to-control ratio of 1:4 by age, sex, and Elixhauser Comorbidity Index (ECI) that includes known risk factors for new-onset depression after TJA such as alcohol and drug abuse [10-12].

Ethical approval

This study was exempted from ethical approval by our institutional review board as we utilized a de-identified database and there was no interaction with participants.

Outcome measures

The primary outcome was the development of new-onset depression within six months after TJA. The secondary outcomes were the presence of one-year arthroplasty-related complications [i.e., periprosthetic fracture, prosthetic joint infection (PJI)] and revision. Only those complications occurring after the diagnosis of new-onset depression were included.

Data analyses

Descriptive statistics was used for demographic variables. Continuous variables with non-normal distributions were displayed as median and interquartile range (IQR). Normality was assessed using the Shapiro-Wilk test. Categorical variables were reported as counts and percentages. Continuous and categorical variables were then respectively compared using the Wilcoxon test and Chi-squared test between study and control groups. The median unemployment rate for each cohort was reported and compared with the Wilcoxon test for each study-control group pair.

The prevalence of new-onset depression within six months of TJA was investigated by stratifying income into five median family income levels (≤$20,000, $20,001-$50,000, $50,001-$80,000, $80,001-$110,000, and >$110,000) based on the class-income breakdown by the Pew Research Center [17], divided according to the U.S. tax brackets for heads of households [18], and reported. Furthermore, the income categories were combined into either lower (≤$50,000) or higher income (>$50,000), with the cut-off value approximated by the class-income breakdown reported by the Pew Research Center [17]. Logistic regressions of each of the binary-matched THA and TKA pairs were conducted to analyze the odds ratio (OR) with 95% CIs for being in the low-income strata.

Next, logistic regressions of each of the binary-matched THA and TKA pairs were conducted to obtain the odds of developing one-year postoperative complications. As previously stated, for the study groups, only the complications that occurred after the diagnosis of new-onset depression were counted. Statistical significance was determined by a p-value <0.05. Data analysis was conducted using the statistical software R (University of Auckland) available within the PearlDiver database.

## Results

Baseline demographics

**Figure 1:**
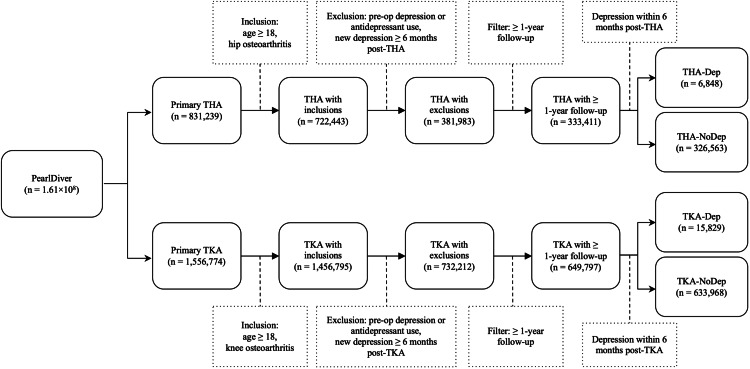
Flow diagram depicting the inclusion of patients in the study Study groups included primary THA and TKA patients with new-onset depression within six months after the surgery (THA-Dep, TKA-Dep). Control groups consisted of those without new-onset depression (THA-NoDep, TKA-NoDep) THA: total hip arthroplasty; TKA: total knee arthroplasty

A total of 333,411 primary THA (6,848 with new-onset depression and 326,563 without) and 649,797 primary TKA (15,829 with new-onset depression and 633,968 without) patients were included (Figure 1). Before matching, the study groups were more likely female in both THA and TKA cohorts compared to the control groups. Other demographic variables of age and ECI were shown to be statistically different but may not be clinically significant. Subsequently, 6,815 THA study group patients were matched to 27,232 THA controls, and 15,796 TKA study group patients were matched to 63,171 TKA controls. Due to the rounding variance of the PearlDiver database’s match function, the results were not exact 1:4 matches, but the age, sex, and ECI of the two THA groups and the two TKA groups were balanced, indicating successful matches (Table 1).

**Table 1:** Pre- and post-match characteristics of THA and TKA patients with new-onset depression within six months (THA-Dep, TKA-Dep) and those without new-onset depression (THA-NoDep, TKA-NoDep) ^+^Wilcoxon test; ^*^Chi-squared test ECI: Elixhauser Comorbidity Index; IQR: interquartile range; THA: total hip arthroplasty; TKA: total knee arthroplasty

Characteristics	THA-Dep	THA-NoDep	P value	TKA-Dep	TKA-NoDep	P-value
Pre-match, n	6,848	326,563		15,829	633,968	
Age, years, median (IQR)	65 (57, 72)	67 (59, 73)	<0.001^+^	66 (59, 72)	68 (61, 73)	<0.001^+^
Female, n (%)	4,208 (61.4)	155,231 (47.5)	<0.001^*^	10,865 (68.6)	338,806 (53.4)	<0.001^*^
ECI, median (IQR)	3 (2, 5)	3 (1, 4)	<0.001^+^	3 (2, 5)	3 (1, 5)	<0.001^+^
Post-match, n	6,815	27,232		15,796	63,171	
Age years, median (IQR)	65 (58, 72)	65 (58, 72)	0.95^+^	66 (59, 72)	66 (59, 72)	0.99^+^
Female, n (%)	4,192 (61.5)	16,755 (61.5)	0.99^*^	10,847 (68.7)	43,388 (68.7)	0.98^*^
ECI, median (IQR)	3 (2, 5)	3 (2, 5)	0.96^+^	3 (2, 5)	3 (2, 5)	0.97^+^

Socioeconomic risk factors for new-onset depression

Among THA patients, 532 (7.8%) patients in the study group and 1,933 (7.1%) patients in the control group were unemployed (p<0.001). Similarly, among TKA patients, 1,232 individuals (7.8%) in the study group and 4,548 individuals (7.2%) in the control group were unemployed (p<0.001).

​​​​Among THA patients, the prevalence of new-onset depression was highest in those with a median family income between $20,001 and $50,000 and lowest in those with a median family income above $110,000. Among TKA patients, the prevalence of new-onset depression was highest in those with a median family income of $20,000 or below and lowest in those with a median family income above $110,000 (Table 2).

**Table 2:** Prevalence of new-onset depression in THA and TKA patients stratified by median family income level Data presented as n (%) THA: total hip arthroplasty; TKA: total knee arthroplasty

Median family income	THA patients with new-onset depression	TKA patients with new-onset depression
$20,000 or below	1,765 of 65,280 (2.7)	4,496 of 124,246 (3.6)
$20,001-$50,000	141 of 4,653 (3.0)	365 of 11,460 (3.2)
$50,001-$80,000	3,649 of 176,137 (2.1)	8,431 of 360,180 (2.3)
$80,001-$110,000	985 of 65,419 (1.5)	2,021 of 120,169 (1.7)
Above $110,000	308 of 21,922 (1.4)	516 of 33,742 (1.5)

Patients who developed new-onset depression (study group) had higher rates and odds of being in the lower-income class compared to those who did not (control group). The THA study group had 1.48 times higher odds (95% CI: 1.40, 1.57; p<0.001) of being in the lower-income class compared to the controls. The TKA study group had 1.66 times higher odds (95% CI: 1.60, 1.73; p<0.001) of being in the lower-income class compared to the controls.

New-onset depression and surgical complications

Patients who developed new-onset depression had higher incidence and odds of one-year periprosthetic fracture, PJI, and revision compared to those who did not. The THA study group had 1.93 times higher odds of periprosthetic fracture (95% CI: 1.50, 2.46; p<0.001), 2.53 times higher odds of PJI (95% CI: 2.11, 3.01; p<0.001), and 1.56 times higher odds of revision (95% CI: 1.31, 1.86; p<0.001) compared to the controls (Table 3). Similarly, the TKA study group had 1.90 times higher odds of periprosthetic fracture (95% CI: 1.34, 2.67; p<0.001), 2.33 times higher odds of PJI (95% CI: 2.06, 2.63; p<0.001), and 1.59 times higher odds of revision (95% CI: 1.37, 1.85; p<0.001) compared to the controls (Table 4).

**Table 3:** One-year arthroplasty-related complications between THA study and control groups CI: confidence interval; OR: odds ratio; THA: total hip arthroplasty

Outcome	THA patients with new-onset depression (n=6,815), n (%)	THA patients without new-onset depression (n=27,232), n (%)	OR (95% CI)	P-value
Periprosthetic fracture	93 (1.4)	194 (0.7)	1.93 (1.50, 2.46)	<0.001
Prosthetic joint infection	203 (3.0)	327 (1.2)	2.53 (2.11, 3.01)	<0.001
THA revision	176 (2.6)	454 (1.7)	1.56 (1.31, 1.86)	<0.001

**Table 4:** One-year arthroplasty-related complications between TKA study and control groups CI: confidence interval; OR: odds ratio; TKA: total knee arthroplasty

Outcome	TKA patients with new-onset depression (n=15,796), n (%)	TKA patients without new-onset depression (n=63,171), n (%)	OR (95% CI)	P value
Periprosthetic fracture	48 (0.3)	101 (0.2)	1.90 (1.34, 2.67)	<0.001
Prosthetic joint infection	410 (2.6)	714 (1.1)	2.33 (2.06, 2.63)	<0.001
TKA revision	241 (1.5)	608 (1.0)	1.59 (1.37, 1.85)	<0.001

## Discussion

We found that primary TJA patients with new-onset depression within six months of surgery had higher unemployment rates and greater odds of being in the lower-income class compared to those without new-onset depression. Additionally, those with new-onset depression had higher rates and odds of one-year periprosthetic fracture, PJI, and revision after TJA. To our knowledge, no studies to date have investigated the association between SEFs, such as employment status and income, and new-onset depression in TJA patients. Our finding of higher prevalence and odds of having new-onset depression among lower-income TJA patients is consistent with prior studies demonstrating associations between low income and higher prevalence of depression in the general population [15]. Future studies may consider other SEFs such as education level and dual eligibility status to understand the comprehensive effects of SDOH as it relates to economic factors on developing new-onset depression.

Our finding of higher rates and odds of 1-year postoperative complications and revisions among TJA patients with new-onset depression is consistent with the current knowledge that TJA patients with pre-existing depression experience poorer TJA outcomes compared to those without [1,4-7]. To our knowledge, this study is the first to examine the association between the presence of new-onset depression and subsequent surgical complications. PJI had the strongest association with new-onset depression after TJA, with the highest OR. This finding is consistent with previous research on THA [10] but differs from a study on TKA [11], which identified periprosthetic fracture as having the strongest association. However, while we considered only those complications occurring after the diagnosis of depression, some may have been symptomatic beforehand, and thus reverse causation cannot be ruled out. For example, early aseptic loosening or deep infection might present as persistent effusion and pain, potentially leading to depression, but could have been officially diagnosed only after the onset of depression. This supports findings from prior studies [10,11] that suggest postoperative complications as risk factors for new-onset depression, rather than the reverse, which we aimed to investigate.

This study has a few limitations, primarily those intrinsic to the PearlDiver database. Due to the limitation of our database in supporting multivariate logistic regression, we adjusted for covariates through matching. Prior studies have identified preoperative anxiety as an important predictor of new-onset depression [10,11]. However, including anxiety in the matching process led to unbalanced groups, so it was not adjusted for in our analysis. Despite this, post-matching analysis showed no significant difference in anxiety rates between the study and control groups. This suggests that SEFs may independently influence the development of new-onset depression. Additionally, data on race was not available in our database, which may be a confounding factor given that depression is more frequent [19] and often underdiagnosed in minority groups [20].

Another confounding factor that may have contributed to postoperative depression is the number of previous operations on the joint, such as arthroscopic or open procedures aimed at delaying the need for joint replacement, which we did not examine in this study. Furthermore, it is possible that those with undiagnosed subclinical depression preoperatively or those with a remote history of depression not recorded in the database [21,22] were inadvertently assigned to the study group, which may weaken the association between new-onset depression and postoperative complications. There is also a lack of specific patient-level data on psychological and clinical factors, such as pain severity and support systems [23], as well as depression severity, which may differentially impact the development of new-onset depression and postoperative outcomes.

Lastly, the potential effects of medications such as antidepressants were not considered in the study. For instance, some selective serotonin reuptake inhibitors (SSRIs) have been associated with an increased risk of falls [24], bone loss [25], and fractures in the elderly, especially among women [26]. Thus, these may have potentially increased the risk of one-year complications for TJA patients with new-onset depression treated with SSRIs and confounded our results. However, other studies have reported reduced pain and improved recovery quality in THA and TKA patients taking duloxetine perioperatively [27,28], and future investigations could explore which antidepressants are most beneficial in the context of new-onset depression. The relationship between new-onset depression and postoperative complications is thus complex and warrants further investigation. This should include the aforementioned confounders as well as other potential complications related to a depressive state that were not examined, such as neurovascular damage, venous thromboembolism [11], and chronic pain [12].

Our findings carry important economic, clinical, and research implications. Studies have shown that TJA patients with depression incur higher hospitalization costs than non-depressed patients [5]. Future studies may perform cost analyses to see if TJA patients with new-onset depression similarly incur higher costs than non-depressed patients, contributing to higher total costs of care in this population. Future studies may also explore additional predictors of depression and outcomes, such as emergency department visits and readmission rates, which could provide valuable insights. Clinicians should educate the patients and engage in discussions with them regarding depressive symptoms pre- and postoperatively and encourage patients to communicate with the office promptly should these symptoms arise.

Patients can also be proactively screened for depression during pre- or postoperative visits with depression surveillance strategies such as routine Patient Health Questionnaire (PHQ)-2 and/or PHQ-9 forms [29] for early detection, being mindful of individuals of low SES statuses. Though this may be sensitive information for the clinician to collect, income ranges may be collected as part of the demographic screening during the patient’s initial visit, as well as other factors related to social determinants of health. Clinicians can then implement appropriate perioperative interventions such as psychiatry referrals and close follow-up to ensure adherence to treatment and rehabilitation. We did not investigate antidepressant use in TJA patients with new-onset depression, but studies found that those with pre-existing depression on antidepressants experienced lower rates of TJA revision [30] compared to non-users. Future studies may explore appropriate management options for TJA patients with new-onset depression and investigate whether treatment of depression is similarly associated with a lower risk of short-term (e.g., 90-day adverse events) or long-term (e.g., revision) complications.

## Conclusions

Our findings showed that new-onset depression is associated with low income. Hence, initial demographic screening during office visits may incorporate questions on income ranges and other social determinants of health to assess patients’ individual risks for this psychiatric complication. Additionally, given the association between new-onset depression and one-year periprosthetic fracture, PJI, and revision, it is important for clinicians to proactively screen patients for depressive symptoms pre- and post-surgery. For patients who exhibit new-onset depressive symptoms, timely psychiatry referrals and close follow-ups may be beneficial. Future studies may investigate whether antidepressant use reduces the risk of postoperative complications in this population.

## Appendices

**Table 5:** CPT, ICD, and USC codes used to define the study groups and postoperative complications CPT: Current Procedural Terminology; ICD-9/10: International Classification of Disease, Ninth/Tenth Revision; USC: Uniform System of Classification; THA: total hip arthroplasty; TKA: total knee arthroplasty

Description	Codes
Antidepressants	USC-64310, USC-64320, USC-64330, USC-64340, USC-64350, USC-64360, USC-64380, USC-64390
Depression	ICM-9: 296.20, 296.21, 296.22, 296.23, 296.24, 296.25, 296.26, 296.30, 296.31, 296.32, 296.33, 296.34, 296.35, 296.36, 296.82, 309.1, 311 ICM-10: F32.0, F32.1, F32.2, F32.3, F32.4, F32.5, F32.8, F32.89, F32.9, F33.0, F33.1, F33.2, F33.3, F33.4, F33.41, F33.42, F33.8, F33.9
Osteoarthritis of hip	ICD-9: 715.15, 715.25, 715.35, 715.95 ICD-10: M16.0, M16.10, M16.11, M16.12, M16.2, M16.30, M16.31, M16.32, M16.4, M16.50, M16.51, M16.52, M16.6, M16.7, M16.9
Osteoarthritis of knee	ICD-9: 715.16, 715.26, 715.36, 715.96 ICD-10: M17.0, M17.10, M17.11, M17.12, M17.2, M17.30, M17.31, M17.32, M17.4, M17.5, M17.9
Total hip arthroplasty	CPT: 27130
Total knee arthroplasty	CPT: 27447
THA periprosthetic fracture	ICD-9: 996.44 ICD-10: M97.9XXA, T84.049A, M97.01XA, M97.02XA, T84.040A, T84.041A
TKA periprosthetic fracture	ICD-9: 996.44 ICD-10: M97.9XXA, T84.049A, M97.11XA, M97.12XA, T84.042A, T84.043A
THA prosthetic joint infection	ICD-9: 996.66 ICD-10: T84.50XA, T84.51XA, T84.52XA
TKA prosthetic joint infection	ICD-9: 996.66 ICD-10: T84.50XA, T84.53XA, T84.54XA
THA revision	CPT: 27134, 27137, 27138
TKA revision	CPT: 27486, 27487
